# Assessing Cancer Knowledge in the General Population of Bisha Province, Saudi Arabia: A Cross-Sectional Study

**DOI:** 10.7759/cureus.75273

**Published:** 2024-12-07

**Authors:** Ahmed Alameer, Sami Mohammed A Alaklabi, Saud G Alshmrani, Malik Alamri, Abdulaziz Faleh S Alshahrani, Obaid F Alharthi, Sirin Dhafer J Alshahrani, Albatool Khaled M Al shahrani, Mohammed Abdulrahman B Alshamrani, Sultan Mofleh S Albogami, Bashayr Mubarak A Alshahrani, Saud Fehaid M Alshahrani, Mohammed Saeed M Alaklbi, Raghad Sayyaf S Alshahrani

**Affiliations:** 1 Department of Breast and Endocrine Surgery, University of Bisha, Bisha, SAU; 2 Department of Surgery, College of Medicine, University of Bisha, Bisha, SAU

**Keywords:** awareness, cancer risk factors, cancer symptoms, general surgery, saudi arabia

## Abstract

Background

Cancer is a major cause of morbidity and mortality worldwide. It is anticipated that the number of new cases in Saudi Arabia will increase yearly as a result of significant changes in lifestyle and population development. There is little to no information or studies concerning cancer awareness or knowledge among the residents of Bisha Province.

Aim

This study set out to determine the degree of cancer awareness among the general population of Bisha Province, focusing on assessing their knowledge level regarding cancer symptoms, diagnosis, and risk factors. The study demonstrated the association between the participants' sociodemographic characteristics and their level of cancer knowledge.

Method

This cross-sectional community survey was carried out in the Kingdom of Saudi Arabia's Bisha Province from August 1, 2024, to October 3, 2024. A total of 402 participants aged 18 and above were selected through a non-probability convenience sampling method. Data were collected by using an online, self-administered, standardized questionnaire.

Results

Awareness levels were generally high, with 243 (60.5%) participants demonstrating excellent awareness levels. However, gaps were observed: only 155 (38.6%) participants showed partial awareness of symptoms and 144 (35.8%) were unaware of self-care practices. Most participants, 274 (68.16%), relied on social media for cancer information. Higher levels of education were shown to be significantly associated with greater awareness. Females demonstrated higher knowledge levels, likely due to female-targeted campaigns, with 118 (78.15%) attending breast cancer awareness events.

Conclusion

The findings suggest the need for more interventional measures to increase population awareness such as targeted educational campaigns, considering demographic differences and diverse communication channels.

## Introduction

Cancer is the second greatest cause of death globally after ischemic heart disease [1]. According to the International Agency for Research on Cancer (IARC), the global cancer burden reached 18.1 million new cases and 9.6 million deaths in 2018 [2]. Also, according to the Global Cancer Observatory, cancer is expected to cause 29.5 million new cases and 16.4 million deaths globally by 2040 [3]. The World Health Organization also reported the number of new cases in the Kingdom of Saudi Arabia for the year 2022 in the organization's vital statistics, which amounted to 28,113 new cases, including 14,745 cases for males and 13,386 cases for females. It also recorded a number of deaths of 13,399 deaths, of which 8,050 deaths were recorded for males and 5,349 deaths for females [4]. Although there are not many cancer cases in Saudi Arabia in comparison to other countries, the number is expected to rise annually as a result of drastic lifestyle changes and the country's expanding population [5]. The importance of early detection appears with increasing cancer survival [6]. Therefore, to achieve this, there is a need to focus on two key components: screening and education for healthcare providers and the public [7]. Cancer screening programs can lead to early diagnosis and intervention, leading to better disease outcomes [4]. Research supports the effectiveness of screening programs in reducing incidence and mortality rates. The American Cancer Society (ACS) recommends annual screening for some malignancies, including colorectal cancer (CRC), starting at age 50. The May 2018 proposal suggests starting CRC screening at the age of 45 [8].

However, studies undertaken in developed and developing nations, including Arab and Middle Eastern countries, revealed that public understanding and awareness of cancer risk factors is low [9]. Raising awareness of cancer is essential for promoting behavior modification and help-seeking. Numerous studies stress the significance of increasing public knowledge of cancer risk factors and symptoms, as well as examining intentions to modify one's lifestyle [10].

Problem statement

It is evident that the burden of cancer continues to grow both locally and internationally, resulting in emotional, physical, and financial strain on families, individuals, the healthcare system, as well as society [11]. Cancer remains a leading cause of morbidity and mortality worldwide, with its burden steadily increasing across all regions, including the Middle East [12]. In Saudi Arabia, where cancer incidences are projected to rise significantly in the coming decades, early detection and prevention through awareness and education are critical [13]. However, there is a substantial knowledge gap among the general population about cancer risk factors, symptoms, and the importance of early screening and preventive measures. Truth be told, the lack of all this knowledge is a major lead to misconception [14]. The public’s understanding of the disease (cancer) and its risk factors is extremely imperative to its management and prevention. The Bisha Province has not been thoroughly investigated for its residents' understanding and awareness of cancer. This gap in knowledge could lead to delayed diagnoses, inadequate healthcare-seeking behavior, and ultimately poorer health outcomes. Most of the studies that have been published were limited to either some specific risk factors and cancer screening or limited to specific types of cancer such as colorectal cancer or breast cancer. There is little to no information or studies concerning cancer awareness or knowledge among the residents of Bisha Province. Hence, there is a need to explore this gap. This study aims to assess the level of knowledge regarding various aspects of cancer among the general population of Bisha Province, focusing on assessing their knowledge level regarding cancer symptoms, diagnosis, and risk factors. The study demonstrated the association between the participants' sociodemographic characteristics and their level of cancer knowledge.

## Materials and methods

This cross-sectional community survey was carried out in the Kingdom of Saudi Arabia's Bisha Province from August1, 2024, to October 3, 2024. The primary purpose of the research was to investigate cancer awareness among the general people in this region.

Using the Cochrane sample size calculation, the sample size was established with a 5% margin of error and a 95% confidence level. Although the lowest acceptable sample size was 384 students, we decided to employ a larger sample of 402 students to improve the reliability.

The research included participants aged 18 and above who had resided in Bisha Province for at least six months. Both males and females were included, with the exception of medical professionals (such as medical students and healthcare practitioners), who were excluded to reduce bias due to their supposedly superior baseline knowledge of cancer. Those who did not fit the inclusion criteria or chose not to participate were also not included.

Participants were selected using a non-probability convenience sampling method. To reach the number of individuals, data were collected using a range of digital platforms such as Facebook, Twitter, Telegram, and WhatsApp.

Data were collected using an online, self-administered, standardized questionnaire. The researchers modified a previous, validated study's questionnaire to better suit the target population of Bisha province [15]. To ensure authenticity and consistency, a professional translator translated the original English text into Arabic and back.

The questionnaire included two sections:

Information about sociodemographics: this section collected information from participants on their age, gender, marital status, education level, place of residence, nationality, occupation, and income.

Cancer knowledge: this portion included multiple-choice questions meant to test participants' cancer knowledge, including topics such as disease formation, symptoms, diagnosis, risk factors, lifestyle, therapies, and patient care.

Google Forms (Google LLC, Mountain View, CA, USA) was used to collect the data, which was subsequently exported to Microsoft Excel 2010 (Microsoft Corporation, Redmond, WA, USA) for initial data processing. Statistical analysis was illustrated throughout using the Statistical Package for Social Sciences (SPSS) Version 28 (IBM Corp., Armonk, NY, USA). The participants' sociodemographic traits and knowledge levels were summarized using descriptive statistics such as frequency distributions and percentages.

Chi-square tests were used to check the association between sociodemographic factors and knowledge levels, with a p-value of less than 0.05 indicating statistical significance. Based on their responses to the questionnaire's questions about their level of knowledge, the study divided the participants into three groups: low, acceptable, and excellent. The division into these groups is based on the number of points they obtained from the questionnaire in 14 questions: each complete answer gets 3 points, an incomplete but not wrong answer gets 1 point, and a wrong answer or I don't know gets no points. Therefore, the maximum possible score was 42, which was divided into three categories: less than 50% (<20 points) (low), 50%-69% (21-29 points) (acceptable), and 70%-100% (30-42 points) (excellent).

Due to the temporary suspension of the University of Bisha Institutional Review Board (IRB), the King Abdullah Hospital Ethical Committee (E-CTS REF No. BIS-24-00025-11112024) provided ethical approval for the project. Prior to participation in the study, all individuals gave their informed consent. The participants were informed of the study's objectives and assured that they might withdraw at any time with no penalty. Confidentiality and anonymity were strictly maintained, ensuring that all personal information provided by the participants was secured and used exclusively for research purposes.

## Results

The study included a total of 402 participants (Table 1). The majority of participants were aged 18-30 years, 298 (74.13%), indicating that most of the respondents were young adults. Those aged 31-40 years accounted for 62 (15.42%), while participants aged 41-50 years and above 50 years represented smaller proportions, 26 (6.47%) and 16 (3.98%), respectively. Regarding gender, females constituted half of the participants, 217 (53.98%), with males accounting for 185 (46.02%). Most participants were Saudi nationals, 391 (97.26%), with only 11 (2.74%) being non-Saudi. Regarding marital status, the majority were single, 261 (64.93%), while 121 (30.10%) were married, 17 (4.23%) were divorced, and 3 (0.75%) were widowed. This distribution is consistent with the fact that a significant proportion of the sample was younger and thus more likely to be unmarried.

**Table 1:** Characteristics of the participants. *Significant (p ≤ 0.05). **Non-significant (p > 0.05).

Parameter	Category	Frequency	Percent(%)	Chi-square test (N=492)	p-Value
Age	18-30 years	298	74.1	8.726	0.726*
	31-40 years	62	15.4		
	41-50 years	26	6.5		
	More than 50 years	16	4.0		
Gender	Male	185	46.0	36.325	0.001**
	Female	217	54.0		
Nationality	Saudi	391	97.3	4.39	0.356*
	Non-Saudi	11	2.7		
Marital status	Married	121	30.1	13.521	0.332*
	Single	261	64.9		
	Divorced	17	4.2		
	Widow	3	0.8		
Educational level	Bachelor	237	59.0	35.054	0.004**
	Diploma	44	10.9		
	Postgraduate studies	21	5.2		
	High school or less	100	24.9		
Job status	Employee	113	28.1	28.21	0.03**
	Unemployed	86	21.4		
	Student	174	43.3		
	Retired	13	3.2		
	Freelance	16	4.0		
Monthly income	Less than 5,000 SAR (1,333 USD) per month	266	66.2	16.762	0.159*
	5,000-10,000 SAR (1,330-2,660 USD) per month	62	15.4		
	10,000-15,000 SAR (2,660-3,990 USD) per month	47	11.7		
	More than 15,000 SAR (3,990 USD) per month	27	6.7		
Place of residence	Bisha Governorate	295	73.4	4.043	0.4*
	Villages of Bisha Governorate	107	26.6		

Educationally, the largest proportion of participants had a bachelor's degree, 237 (58.96%), while 100 (24.88%) had a high school education or less. Those with diplomas and postgraduate studies accounted for 44 (10.95%) and 21 (5.22%), respectively. These data suggest that the majority of the participants were relatively well-educated, with a significant portion having completed university education. In terms of occupations, 174 (43.28%) were students, 113 (28.11%) were employed, and 86 (21.39%) were unemployed. A small number of participants were retired, 13 (3.23%), or freelancers, 16 (3.98%). Most participants, 266 (66.17%), earned less than 5,000 SAR (1,330 USD) per month, reflecting a lower-income demographic, which is consistent with the high percentage of students and young participants. A smaller number of participants, 62 (15.42%), fell within the income bracket of 5,000-10,000 SAR (1,330-2,660 USD), followed by 47 (11.69%) in the income bracket of 10,000-15,000 SAR (2,660-3,990 USD) and 27 (6.72%) in the income bracket of above 15,000 SAR (3,990 USD). The majority of participants were residents of Bisha Governorate, 295 (73.38%), while 107 (26.62%) resided in the villages surrounding Bisha.

Different sources of cancer information (Table 2) included social media, health practitioners, educational institutions, and other means. The majority of respondents, 274 (68.16%), relied on social media, while only 142 (35.32%) used health practitioners as a source. Educational institutions and campaigns showed a similar trend, with 121 (30.1%) and 119 (29.6%) of the population considering them as sources of information. However, the "Others" category is the least used, with only 15 (3.73%) of the respondents turning to alternate sources.

**Table 2:** Source of information. *Significant (p ≤ 0.05). **Non-significant (p > 0.05).

Source of information	Yes	No	Chi-square test (N=402)	p-Value
Social media	274 (68.2%)	128 (31.8%)	7.471	0.113
Health practitioners	142 (35.3%)	260 (64.7%)	4.782	0.31
Educational institutions	121 (30.1%)	281 (69.9%)	9.254	0.055
Campaigns	119 (29.6%)	283 (70.4%)	9.369	0.053
Others	15 (3.7%)	387 (96.3%)	8.818	0.066

Despite the high use of social media, the chi-square test indicates that none of the sources showed statistical significance (p-values > 0.05), meaning that no specific source stands out as being more effective or impactful in disseminating cancer-related information.

In questions concerning cancer awareness, only 17 (4.23%) of participants reported having been diagnosed with cancer, while the overwhelming majority, 385 (95.77%), had not (Table 3). However, 107 (26.62%) of respondents indicated that someone in their family had been diagnosed with cancer, reflecting a moderate level of cancer exposure among the participants.

**Table 3:** Cancer-related questions. *Significant (p ≤ 0.05). **Non-significant (p > 0.05).

Cancer-related questions	Category	Frequency	Percent (%)	Chi-square test	p-Value
Have you ever been diagnosed with cancer?	Yes	17	4.2	5.108	0.276*
	No	385	95.8		
Has anyone in your family been diagnosed with cancer before?	Yes	107	26.6	25.907	0.001**
	No	264	65.7		
	Not sure	31	7.7		
Have you ever attended a cancer-related event (e.g., breast cancer awareness, prostate cancer awareness, colon cancer awareness)?	Yes	151	37.6	18.242	0.001**
	No	251	62.4		
If yes, what was it?	Breast cancer event	118	78.1	32.314	0.645
	Colon cancer event	15	10.0		
	Prostate cancer event	11	7.3		
	Others	7	4.6		
If yes, when was it?	Less than a year ago	50	33.1	13.149	0.358
	1-2 years ago	56	37.1		
	2-5 years ago	31	20.5		
	More than 5 years ago	14	9.3		

Regarding cancer awareness events, 151 (37.6%) of participants had attended a cancer-related event, while 251 (62.4%) had not. Among those who had attended such events, the majority were involved in breast cancer awareness events, 118 (78.15%), followed by colon cancer events, 15 (10.0%), prostate cancer events, 11 (7.3%), and other types of events, 7 (4.6%). Finally, when asked about the timing of their participation in cancer-related events, 50 (33.11%) had attended an event less than a year ago, 56 (37.09%) attended an event one to two years ago, 31 (20.53%) attended an event two to five years ago, and 14 (9.27%) attended an event more than five years ago. These results suggest that while cancer awareness is present, there is increased engagement and participation in cancer-related activities.

As shown in Table 4, we modified the findings and rearranged them into three awareness levels. This was done to make it easier to understand the data, which showed 93 (23.13%) with low awareness, 66 (16.41%) with acceptable awareness, 243 (60.45%) with excellent awareness.

**Table 4:** Sociodemographic parameters and levels of awareness.

Parameter	Category	Frequency	Percent %	<20 point, <50%, low awareness	21-29 points, 50%-69%, acceptable awareness	30-42 points, 70%-100%, excellent awareness
Age	18-30 years	298	74.129	69 (23.2%)	48 (16.1%)	181 (60.7%)
	31-40 years	62	15.423	11 (17.7%)	14 (22.6%)	37 (59.7%)
	41-50 years	26	6.468	7 (26.9%)	2 (7.7%)	17 (65.4%)
	More than 50 years	16	3.98	6 (37.5%)	2 (12.5%)	8 (50.0%)
Gender	Male	185	46.02	58 (31.4%)	41 (22.2%)	86 (46.5%)
	Female	217	53.98	35 (16.1%)	25 (11.521%)	157 (72.4%)
Nationality	Saudi	391	97.264	91 (23.3%)	66 (16.880%)	234 (59.8%)
	Non-Saudi	11	2.736	2 (18.2%)	0 (0.00%)	9 (81.8%)
Marital status	Married	121	30.1	30 (7.54%)	21 (17.355%)	70 (17.59%)
	Single	261	64.925	56 (14.07%)	39 (14.943%)	166 (41.71%)
	Divorced	17	4.229	6 (1.51%)	5 (29.412%)	6 (1.51%)
	Widow	3	0.746	1 (0.25%)	1 (33.333%)	1 (0.25%)
Educational level	Bachelor	237	58.955	44 (18.6%)	32 (13.5%)	161 (67.9%)
	Diploma	44	10.945	17 (38.6%)	9 (20.5%)	18 (40.9%)
	Postgraduate studies	21	5.224	7 (33.3%)	7 (33.3%)	7 (33.3%)
	High school or less	100	24.876	25 (25.0%)	18 (18.00%)	57 (57.0%)
Job status	Employee	113	28.109	30 (26.5%)	24 (21.2%)	59 (52.2%)
	Unemployed	86	21.39	20 (23.3%)	16 (18.6%)	50 (58.1%)
	Student	174	43.284	31 (17.8%)	23 (13.2%)	120 (67.0%)
	Retired	13	3.234	4 (30.8%)	1 (7.7%)	8 (61.5%)
	Freelance	16	3.98	8 (50.0%)	2 (12.5%)	6 (37.5%)
Monthly income	Less than 5,000 SAR per month	266	66.169	51 (19.2%)	41 (15.4%)	174 (65.4%)
	5,000-10,000 SAR per month	62	15.423	20 (32.3%)	8 (12.9%)	34 (54.8%)
	10,000-15,000 SAR per month	47	11.692	13 (27.7%)	13 (27.7%)	21 (44.6%)
	More than 15,000 per month	27	6.716	9 (33.3%)	4 (14.8%)	14 (51%)
Residency	Bisha Governorate	295	73.383	73 (24.7%)	47 (15.9%)	175 (59.3%)
	Villages of Bisha Governorate	107	26.617	20 (18.7%)	19 (17.8%)	68 (63.5%)

In terms of awareness levels, females displayed higher percentages of excellent awareness than males, 72.4% for females versus 46.5% for males. Nationality distribution showed that the vast majority were Saudi nationals (97.3%), with a small proportion of non-Saudis. Non-Saudi nationals displayed higher excellent awareness levels than Saudis, 9 (81.8%) versus 234 (59.8%). Educational levels show that those with postgraduate studies had a notably higher percentage of acceptable awareness, 7 (33.33%), though a significant portion of those with bachelor's degrees showed excellent awareness, 161 (67.9%). Employment status revealed that students formed the largest group, 174 (43.28%), and had the highest percentage of excellent awareness, 120 (67.0%).

Overall, the data indicate that awareness levels vary significantly across sociodemographic groups, with educational level, gender, and employment status and have a clear impact on awareness outcomes.

**Figure 1:**
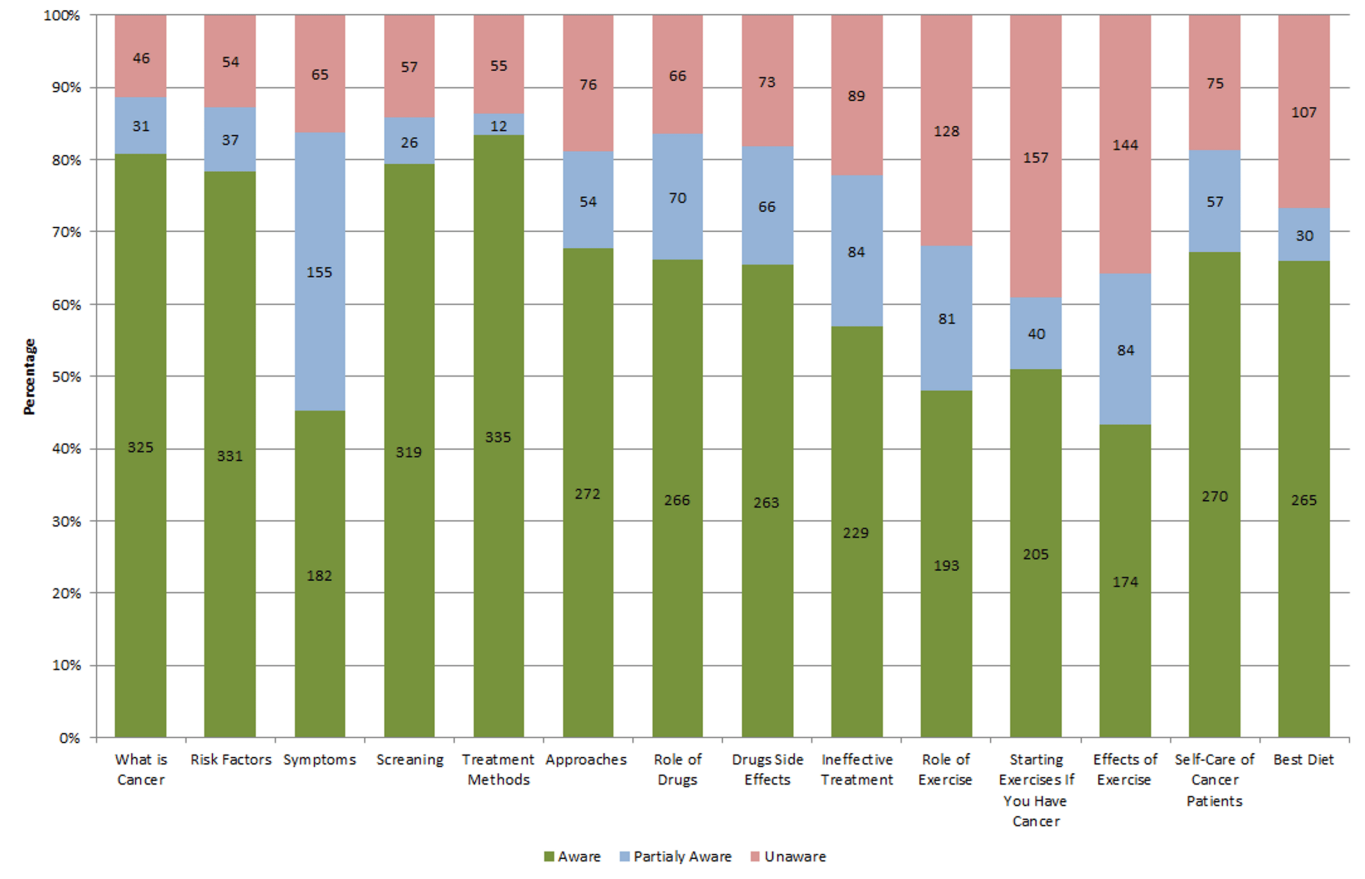
Awareness level for each question. Check the Appendices for more details about the questions

Awareness levels were generally high, especially in areas such as understanding cancer itself, 325 (80.8%), risk factors, 331 (82.3%), and treatment methods, 335 (83.35) (Figure 1).

Partial awareness was moderate in areas such as symptoms, 155 (38.65), and side effects of drugs, 84 (20.9%). Unawareness was significant in certain areas, notably in understanding when to begin physical exercises, 157 (39.1%) and self-care 144 (35.8%). Overall, the data suggest a good level of awareness among participants, though specific areas such as symptom recognition, exercise timing, and self-care need further education.

## Discussion

Cancer is a global health crisis resulting in a significant emotional, physical, and financial burden to individuals and their families. Cancer impact is felt in all regions, including the Middle East. Despite advancements in medical treatment, cancer remains a major cause of morbidity and mortality [11,12]. This study assessed the level of cancer knowledge among the general population of Bisha. More than half of the participant, 274 (68.16%), cited social media as their primary source of information regarding cancer. These findings are not surprising, as it was also demonstrated by Ibrahim et al. [16], who reported similar results in which participants used social media as the primary source of information. In our study, younger individuals (18-30 years) showed higher levels of awareness. This could be attributed to social media as it was the highest cited source. This finding demonstrates the effectiveness of social media in improving awareness. A study conducted by Prager et al. [11] highlighted the role of social media in increasing awareness. The study also emphasized the role of accurate dissemination of information as it may lead to unfavorable outcomes, especially in middle-income countries, with 243 (60.44%) of participants showing excellent levels of knowledge. Participants showed partial awareness in areas such as symptoms, 155 (38.6%), and side effects of drugs, 84 (20.9%). Participants were unaware in areas of understanding when to begin physical exercises, 157 (39.1%), and self-care, 144 (35.8%).

These disparities align with a study conducted by Algamdi et al. [14], which shows a good level of awareness regarding cancer but a lack of knowledge of the role of lifestyle and early detection. These findings may reflect a gap in general cancer awareness campaigns. Although have been successful in promoting awareness of cancer among the population, still lack education in these specific areas. Additionally, Al-Azri et al. [17] showed a strong correlation between participants' answers and their educational attainment. They were more likely to recognize cancer risk factors if they had greater education. This aligns with our findings in which notable associations were found between educational level and level of awareness. Those with bachelor’s or postgraduate degrees showed significantly higher levels of cancer awareness. Interestingly, these findings were found to be the opposite in Algamdi et al.’s study, in which higher educated participants in the study had low-risk factor knowledge scores. The study found a strong correlation between risk factor knowledge and the identification of a family history of cancer. Individuals with a family history of cancer were generally more aware of cancer-related topics than those without such a history. The study conducted by Poudel and Naomi reported the same findings [18]. Sociodemographic factors, particularly education and gender, play a significant role in cancer awareness. In our study, gender shows a statistical significance in which females show higher knowledge than males. This is also seen in the findings of multiple studies [14,19-21]. These findings suggest that females tend to have better knowledge due to the widespread of female-targeted campaigns. This aligns with our finding in that most of the participants were involved in breast cancer awareness events, accounting for 118 (78.1%). While breast cancer awareness is well-established, as our study reveals, knowledge of other cancers such as colorectal, 15 (10.0%), and prostate cancer, 11 (7.29%), remains limited. Public health authorities should develop targeted campaigns for these cancers to ensure a well-informed population.

This study has some limitations. The use of a convenience sampling method may limit the generalizability of the findings to the entire population of Bisha Province. It was an online questionnaire and most of the online users were youths (75%), and that was reflected clearly in this study. Additionally, this approach may have introduced biases that restrict the generalizability of the findings. Moreover, distributing the questionnaire via social media platforms such as WhatsApp could have excluded individuals with lower educational backgrounds and socioeconomic statuses.

## Conclusions

This study provides insight into the general public's awareness of cancer in Saudi Arabia's Bisha Province. The findings indicate that although the majority of participants possess a solid awareness of cancer, including its risk factors and available treatments, there are still notable knowledge gaps. Numerous participants had trouble identifying the signs of cancer, comprehending the importance of exercise, and implementing self-care routines, indicating areas that require targeted attention.

It was determined that the main source of knowledge about cancer was social media. In conclusion, although the region has a comparatively high level of general cancer awareness, focused educational initiatives are required to fill in the knowledge gaps, particularly about symptoms and self-care. These programs should use a variety of communication channels and take into account demographic differences to enhance their effectiveness in raising awareness and promoting early detection and prevention strategies.

## Appendices

Consents and Questionnaire

Consent Form

We are medical students from Bisha University conducting a research study titled "Assessing Cancer Knowledge in the General Population of Bisha Province, Saudi Arabia." We kindly request your participation in this study by answering the following questionnaire.

Please be assured that your responses will remain confidential, and your identity will not be disclosed. Participation is entirely voluntary, and you have the right to withdraw at any time without any consequences.

Please click "Yes" below to indicate your agreement to participate.

Yes.      ⃝  No.        ⃝

Do not hesitate to contact us if you need more information or if you have any questions.

Through

Phone N: 0500065300.                 Email: Samealaklbe2@gmail.com

**Table 5:** Questionnaire form. This questionnaire is adapted from Pereira et al. [15] under the Creative Commons Attribution 4.0 International License (CC BY 4.0). The license permits unrestricted use, distribution, and adaptation of the material, provided appropriate credit is given to the original authors. For more details on the license, visit https://creativecommons.org/licenses/by/4.0/

Question	Options
Age:	___ years
Gender:	a) Male. b) Female.
Marital status:	a) Single. b) Married. c) Divorced. d) Widowed.
Educational level:	a) Secondary education or less. b) Diploma. c) Bachelor’s degree. d) Master’s degree or higher.
Occupation:	a) Unemployed or retired. b) Student (non-medical field). c)Employed (non-medical field). d) Freelance.
Income per month:	a) Less than 5,000 SAR per month. b) 5,000–10,000 SAR per month. c) 10,000–15,000 SAR per month. d) More than 15,000 SAR per month.
Nationality:	a) Saudi. b) Non-Saudi.
Place of residence:	a) Urban (City). b) Rural (Village).
History of cancer:	a) Yes. b) No.
Family history of cancer:	a) Yes. b) No. c) Not sure.
Have you ever attended cancer-related events (e.g., breast cancer awareness, prostate cancer awareness, colon cancer awareness):	a) Yes. b) No.
If yes, what was it?	a) Breast cancer event. b) Prostate cancer event. c) Colon cancer event. d) Other: ____.
When was it?	a) Less than 1 year. b) 1-2 years. c) 2-5 years. d) More than 5 years.
Source of information about cancer:	a) Social media. b) Health professionals. c) Educational institutions. d) Campaigns. e) Others.
What is cancer?	a) Cancer is an illness that strikes older adults because their bodies' defenses are weakened. b) Cancer is the result of abnormal cell proliferation that invades organs and tissues and has the potential to spread to other bodily parts. c) Cancer is a disease that is only inherited and is passed down from parents to their offspring. d) I'm not sure.
Which risk factors impact the development of cancer the most?	a) Low family income and low levels of education. b) Genetic predisposition and lifestyle choices (smoking, drinking, eating poorly, and not exercising). c) Obesity and age beyond 65. d) I'm not sure.
Which symptoms are most prevalent among cancer patients?	a) The symptoms of cancer differ depending on the body part that is impacted. b) A cancer patient experiences no emotions. c) Night sweats, exhaustion, chills, and weight loss are the most typical symptoms. d) I'm not sure.
Which strategy is the most effective for cancer early detection?	a) A urine test. b) Routine physical examinations, blood tests, imaging tests, and medical exams. c) Stress test on a treadmill. d) I'm not sure.
Which cancer-treating procedures are most frequently used?	a) Surgery, radiation, chemotherapy, and occasionally immune treatment. b) Since cancer is a genetic condition, there is no cure. c) Replacing hormones. d) I'm not sure.
Which cancer treatment approaches can improve patients' quality of life?	a) Give up your job and family, as well as any medication, surgery, or extended, complete bed rest. b) Medication therapy and, if required, surgery. c) Modification of lifestyle, avoidance of risk factors that worsen the disease, and, if required, medication and surgery. d) I'm not sure.
The role of drug treatment for cancer is:	a) To improve the patient’s physical and psychological condition. b) To avoid infectious diseases. c) To prevent the diseased cells from growing and multiplying rapidly, out of control, and aggressively. d) I don’t know.
What are the most typical adverse effects of the medications used to treat cancer?	a) Fatigue, nausea, vomiting, diarrhea, sleeplessness, and hair loss. b) Pain in their joints, muscles, and bones. c) Weight gain, heightened hunger, and enhanced sexual performance. d) I'm not sure.
Examine one of the effects of ineffective cancer treatment:	a) A rise in fatigue, weakness, and depression. b) The body's defenses are weakened, resulting in increasing symptoms and an increased chance of mortality. c) The manifestation of additional illnesses, including diabetes. d) I'm not sure.
Regarding cancer patients' physical activity:	a) Cancer patients should never exercise because it raises their risk of dying. b) Once the patient is clinically stable, physical activity ought to be incorporated into the therapy plan. c) Exercise is a component of the treatment since it helps alleviate symptoms and increase muscle strength and physical conditioning. d) I'm not sure.
Cancer patients should:	a) Start exercising as soon as they are diagnosed. b) Honour the patient's needs, which the medical staff will assess and prescribe on an individual basis. c) Since men and women have similar levels of physical conditioning, physical activity should be the same for both sexes at the same age. d) I'm not sure.
What are the main benefits of exercise for people with cancer?	a) Blood glucose maintenance and a reduction in body fat and resting heart rate. b) Higher blood sugar, elevated cholesterol, and elevated pulse rate. c) Enhances quality of life, reduces weariness and depression, and fortifies the body's defense cells. d) I'm not sure.
When it comes to cancer patients' self-care, it's critical to understand that:	a) Patients should be aware of their condition. b) Patients and their families should be informed about the illness because it can aid with therapy and enhance the patients' quality of life. c) Understanding how the disease progresses and is treated is not crucial; that is the duty of the medical staff. d) I'm not sure.
What is the ideal diet for people with cancer?	a) A diet rich in fruits, vegetables, whole grains, fiber, and vitamins. b) A typical diet that is salty and sour to increase hunger. c) A diet reduced in fat and salt. d) I'm not sure.
Examine one of the effects of insufficient cancer treatment:	a) A rise in fatigue, weakness, and depression. b) The body's defenses are weakened, resulting in increasing symptoms and an increased chance of mortality. c) The manifestation of additional illnesses, including diabetes. d) I'm not sure.
What are the main benefits of exercise for people with cancer?	a) Blood glucose maintenance and a reduction in body fat and resting heart rate. b) Higher blood sugar, elevated cholesterol, and elevated pulse rate. c) Enhances quality of life, reduces weariness and depression, and fortifies the body's defense cells. d) I'm not sure.

## References

[1] (2018). Global, regional, and national age-sex-specific mortality for 282 causes of death in 195 countries and territories, 1980-2017: a systematic analysis for the Global Burden of Disease Study 2017. Lancet.

[2] Bray F, Ferlay J, Soerjomataram I, Siegel RL, Torre LA, Jemal A (2018). Global cancer statistics 2018: GLOBOCAN estimates of incidence and mortality worldwide for 36 cancers in 185 countries. CA Cancer J Clin.

[3] Ferlay J, Ervik M, Lam F, Colombet M, Mery L, Pineros M: (2019). Global Cancer Observatory: Cancer Today. International Agency for Research on Cancer.

[4] (2021). World Health Organization. Cancer. https://www.who.int/news-room/fact-sheets/detail/cancer.

[5] Althubiti MA, Nour Eldein MM (2018). Trends in the incidence and mortality of cancer in Saudi Arabia. Saudi Med J.

[6] Schiffman JD, Fisher PG, Gibbs P (2015). Early detection of cancer: past, present, and future. Am Soc Clin Oncol Educ Book.

[7] International Agency for Research on Cancer (IARC (2018). International Agency for Research on Cancer. Section of Early Detection and Prevention. https://www.iarc.who.int/.

[8] American Cancer Society (2018). Updates Colorectal Cancer Screening Guideline. https://www.cancer.org/health-care-professionals/american-cancer-society-prevention-early-detection-guidelines/colorectal-cancer-screening-guidelines.html.

[9] Breslow RA, Sorkin JD, Frey CM, Kessler LG (1997). Americans' knowledge of cancer risk and survival. Prev Med.

[10] Smith SG, Osborne K, Tring S, George H, Power E (2016). Evaluating the impact of a community-based cancer awareness roadshow on awareness, attitudes and behaviors. Prev Med.

[11] Prager GW, Braga S, Bystricky B (2018). Global cancer control: responding to the growing burden, rising costs and inequalities in access. ESMO Open.

[12] Arafa MA, Rabah DM, Farhat KH (2020). Rising cancer rates in the Arab World: now is the time for action. East Mediterr Health J.

[13] Mahdi H, Mula-Hussain L, Ramzi ZS (2022). Cancer burden among Arab-world females in 2020: working toward improving outcomes. JCO Glob Oncol.

[14] Algamdi M, Gonzales A, Farah E (2021). Awareness of common cancer risk factors and symptoms in Saudi Arabia: a community-based study. Asian Pac J Cancer Prev.

[15] Pereira Junior M, Santos RZ dos, Ramos AP, Andrade A, Santos LRM dos, Benetti M (2018). Development and psychometric validation of cancer-Q: questionnaire about cancer patient’s knowledge of their disease. Rev Bras Cancerol.

[16] Ibrahim N, Almuhsin A, Alshaibani A (2020). Cancer awareness in Saudi Arabia: a cross-sectional population-based observational study. Glob J Med Therap.

[17] Al-Azri M, Al-Rasbi K, Al-Hinai M, Davidson R, Al-Maniri A (2014). Awareness of risk factors for cancer among Omani adults--a community based study. Asian Pac J Cancer Prev.

[18] Poudel K, Sumi N (2017). Awareness of cancer in Asian countries-a review of the literature. J Compr Nurs Res.

[19] Ravichandran K, Mohamed G, Al-Hamdan NA (2010). Public knowledge on cancer and its determinants among Saudis in the Riyadh Region of Saudi Arabia. Asian Pac J Cancer Prev.

[20] Qassim S, Al-Hariri Y, Shanableh S, Farajallah A, Boura F (2018). Awareness level of cancer warning signs and its determinants among university students in UAE. J Pharm Sci Res.

[21] Feizi A, Kazemnejad A, Hosseini M, Parsa-Yekta Z, Jamali J (2011). Assessing awareness level about warning signs of cancer and its determinants in an Iranian general population. J Health Popul Nutr.

